# Taxation and the Voluntary Hospitals

**Published:** 1914-12-12

**Authors:** 


### The Hospital

The Workers' Newspaper of Administrative Medicine and Institutional Life, Administration, National Insurance and Health.

No. 1486, Vol. LVII. SATURDAY, DECEMBER 12, 1914 PRICE ONE PENNY.

### TAXATION AND THE VOLUNTARY HOSPITALS.

The time has surely come when the managers of the voluntary hospitals and their supporters should combine to arouse the Government of this country to the continuous ill-treatment and illiberality which has characterised and is at present characterising their action, through legislation, towards the voluntary hospitals. When National Insurance was introduced the members of the Government responsible for the Act and for its administration over and over again declared with emphasis that adequate medical treatment was to be provided through its means to a multitude of persons. As we have pointed out, before the Acts became law, and since, there was no money provided to defray the cost of adequate medical treatment for all insured persons. When the outcry on this head became irresistible and the evidence proved damning, the representatives of the Government stated that the Acts were only intended to provide adequate medical treatment for ordinary cases such as a general practitioner could treat in the ordinary way. Not one word was said, by any politician responsible, of the financial injury which must result to the voluntary hospitals from first exciting false hopes that free in-patient treatment would be granted to every insured person needing it, thereby creating a new and largely increased demand for such provision. Could any policy or any propaganda more unwise or more unjust to the voluntary hospitals of this country be conceived than this?

Again, we have called attention more than once recently to the question of duty-free rectified spirit in hospitals. In an article we published in our issue of November 21, page 181, we answered certain objections which had been raised officially to the argument against the proposal which justice has advocated—namely, that the voluntary hospitals should be allowed duty-free spirit in the manufacture of tinctures. The objections related to the distinction drawn by the Inland Revenue authorities between duty-free spirit (*a*) in the manufacture of galenicals, in which process the alcohol is not destroyed; and (*b*) in the manufacture of chloroform, ether, etc., in which process the spirit is no longer present as such. We pointed out clearly that the objections which apply to a concession to galenical manufacturers of duty-free spirit, do not and cannot apply to such a concession to the hospitals, because the latter can readily satisfy any conditions the Customs and Excise authorities may impose, whilst the galenicals in hospitals, which we urged should be made up with duty-free spirit, are solely consumed by hospital patients or are used for hospital purposes on the prescriptions of medical men. We therefore urged for the reasons stated, which we believe to be unanswerable, that a joint application should be made to the Inland Revenue authorities by British voluntary hospitals, because unity would prove to be strength in the present circumstances. As so often happens where an English Government has to do with English traders, the English manufacturer is placed at a positive disadvantage with his foreign competitors, and at the same time further burdens are imposed on British hospitals amounting to many pounds per annum. Once again the selection of beer as an article for taxation in connection with the moneys required for war purposes hits the hospitals unduly hard, first because [as our Special Commission of experts proved in their report * on the subject which has become a classic] to residents in the British Islands beer is a staple food product of real value for climatic reasons and others. Beer, though not in large quantities, is supplied at most hospitals, and we have before us an invoice where the tax on a quarter cask (nine gallons) of B.A. ale is 5s., so that the sum which has to be paid for nine gallons of ordinary ale is now 14s. instead of 9s. paid previously.

We have called attention to the foregoing instances of the utter disregard by the Government of the claims of the voluntary hospitals (*a*) when new legislation is enacted, (*b*) when British and foreign traders are in competition, (*c*) when the hospitals are helping the State by giving free relief to Government cases, as well as to ordinary members of the population, and (*d*) when new taxes are imposed. Our cases are those which have arisen within the immediate past, and at least three of them constitute a clear wrong to the voluntary hospitals if equity is still to hold its own in matters of

* *Vide* The Hospital, April 20, 1909.

legislation and the treatment of institutions by Government authorities. The sum of money involved in regard to the amount of free in-patient treatment at the voluntary hospitals given to insured persons without any compensation, already amounts to hundreds of thousands of pounds, to which must be added the further sums payable owing to the enforcement of revenue charges upon hospitals in the case of rectified spirit, and to meet the heavy extra war-tax upon beer consumed entirely in the hospitals. Having regard to the enormous service which the voluntary hospitals have rendered to the Government since the passing of the National Insurance Acts, in addition to their invaluable aid to the Poor-Law authorities from time immemorial, we venture to hope the managers of the hospitals will combine to bring the matter to the notice of the authorities immediately concerned without hesitation or delay. Seeing that we have now no parties or politics in Parliament, it would be a gracious act of the Chancellor of the Exchequer, who is personally under a lifelong debt to the voluntary hospitals for the help they have rendered to him in regard to the successful enforcement of National Insurance, that he should take these matters promptly into his personal consideration and spontaneously declare himself upon the various points we have ventured to raise.

